# Protective effect of crocin on bisphenol A - induced spatial learning and memory impairment in adult male rats: Role of oxidative stress and AMPA receptor

**DOI:** 10.22038/ijbms.2020.41097.9714

**Published:** 2020-09

**Authors:** Faezeh Vahdati Hassani, Elaheh Masjedi, Hossein Hosseinzadeh, Zeinab Bedrood, Khalil Abnous, Soghra Mehri

**Affiliations:** 1 Department of Pharmacodynamics and Toxicology, School of Pharmacy, Mashhad University of Medical Sciences, Mashhad, Iran; 2 Pharmaceutical Research Center, Pharmaceutical Technology Institute, Mashhad University of Medical Sciences, Mashhad, Iran

**Keywords:** AMPA, Anti-oxidant, Bisphenol A, Crocin, Crocus sativus L., NMDA

## Abstract

**Objective(s):**

Bisphenol A (BPA), a xenoestrogenic endocrine disrupting agent, is widely used in the production of polycarbonate plastics and has potential adverse effects on the developing nervous system, memory and learning abilities. The protective effect of the crocin, an important active constituent in *Crocus sativus* L, on memory impairment induced by BPA in rat was determined through evaluation of oxidative stress and the level of NMDA (N-methyl-D-aspartate receptors) and AMPA (α-amino-3-hydroxy-5-methyl-4-isoxazole-propionicd acid) receptors.

**Materials and Methods:**

Rats were orally treated with BPA (100 mg/kg) or sesame seed oil in control group for 28 days. Crocin (10, 20, and 40 mg/kg, IP) was administrated in BPA-orally treated groups for 28 days. Memory and learning functions were evaluated by Morris water maze. The level of malondialdehyde (MDA) and glutathione (GSH) contents were determined in rat hippocampus. Additionally, the expression of NMDA and AMPA receptors were analyzed using Western blot method.

**Results:**

Administration of BPA significantly reduced memory and learning functions. Crocin significantly protected against learning and memory impairments induced by BPA. BPA administration markedly reduced GSH content and induced lipid peroxidation, while crocin was able to increase GSH content in rat hippocampus. The expression of NMDA receptor did not change in BPA-treated rats, while the significant reduction in AMPA receptor expression was observed. Moreover, crocin (20 mg/kg) significantly elevated the expression of AMPA receptor.

**Conclusion:**

Crocin recovered spatial learning and memory defects induced by BPA in part through anti-oxidant activity and modulation the expression of AMPA receptor in rat hippocampus.

## Introduction

Several areas of the brain have important roles in brain processes associated with declarative, episodic, and recognition memory (1). Different studies confirm gonadal hormones affect the development of the brain and brain function during adulthood. Estrogen influences many processes in hippocampal formation that are important for learning, memory and emotion (2). Growing evidence has indicated that gonadal steroids also exert profound effects on the development and differentiation of neurons and synaptic plasticity in the adult brain (3-6). Exposure of hippocampal slices of male Wistar rats to the letrozole as an aromatase inhibitor acutely decreased the magnitude of postsynaptic currents following activation of N-methyl-D-aspartate (NMDA) receptor and inhibited long-term potentiation (LTP) which is dependent to NMDA receptor in the dentate gyrus. It shows that estradiol significantly reinforces NMDA receptor dependent transmission and, so, synaptic plasticity markedly is facilitated (7). In addition NMDA receptors have critical role on memory, the acquisition of spatial learning and induction of long-term synaptic plasticity (8, 9). Also, the expression of synaptic plasticity and LTP can be modulated through another ionotropic glutamate receptor α-amino-3-hydrozy-5-methylisoxazole-4-propionic acid (AMPA) function (10). The hippocampus is considered as a site of damage in several neurological disease and is susceptible to oxidative stress (11). During LTP processes the appropriate function of NMDA receptor at hippocampal synapses is related to maintenance of the intracellular redox state (12). Neurotoxicity is one the important problems following exposure to chemical environmental agents. Neurotoxicity can be manifested as neuropathological, electrophysiological, altered neurocthemical, or behavioural dysfunctions (13).

Bisphenol A (BPA), as the environmental toxic agent can disrupt endocrine function. BPA is produced in great amounts to prepare polycarbonate plastics, epoxy resin in linings for metal food cans and dental sealants. Heat and acidic or basic conditions accelerate BPA release from these products leading to potential human exposure (14). BPA has been found in urine (15), saliva (16), follicular fluid, fetal serum, full-term amniotic fluid (17) and breast milk (18) samples of people. BPA at very low doses similar to high can induce oxidative stress in cells and organs through generation of reactive oxygen species (ROS) and reduction the activity of anti-oxidant enzymes (19-22), as well as the estrogenic effects (23, 24). In addition to weakly interacting with classical nuclear estrogen receptors (ERα and ERβ) as an agonist or antagonist of 17-β estradiol (E2), BPA can inhibit the action of androgen and thyroid hormone (23). BPA suppresses estrogen-dependent hippocampal synapse formation. Therefore, it interferes with the both expression and development of normal sex differences in cognitive function and impairs normal function of hippocampus (25). Studies have reported that short and long term BPA exposure in adult and young adult animals interfere with neural plasticity processes in brain, so block the formation of new memories and change characteristic differences in some non-reproductive behaviours including passive avoidance memory, learning and memory (26, 27). The mentioned effects may be related to the higher susceptibility of the hippocampal synaptic plasticity to BPA (28). Additionally, BPA decreased the number of spine synapses and synaptogenic response of hippocampal and prefrontal area (29-31). 

Crocin, a unique carotenoid, is a crocetin digentiobiose ester with anti-oxidant activities isolated from the dried stigma of flowers of *Crocus sativus* L. (Saffron) (32, 33). Numerous studies have shown hepatoprotective (22), anti-inflammatory (34, 35), antihyperlipidemic, cardioprotective (36-38) and protective effects of crocin against natural toxins and chemicals-induced toxicities (39). Crocin is also an antidepressant agent (40-43) and could improve the expression of proteins regulating activity-dependent forms of synaptic plasticity including LTP, thought to underlie learning and memory (41, 42, 44). Crocin attenuated spatial cognitive dysfunction after chronic cerebral hypoperfusion (45), inhibited the scopolamine-induced unpaired acquisition or performance activity (46), and treated cognitive dysfunction in diabetics rats (47). It is considered that the effects of BPA on memory of adult male rats may be related to the oxidative stress and the alteration in expressions of NMDA and AMPA receptors in the hippocampus. In the present study, we examined the protective effect of crocin on oxidative stress and hippocampus-dependent learning and memory performance following BPA exposure in adult male rats. 

## Materials and Methods

***Chemicals***

Bisphenol A (BPA), (≥99% purity), sesame oil‎, trichloroacetic acid (TCA), bromophenol blue, phenylmethanesulfonyl fluoride (PMSF), sodium fluoride (NaF), sodium orthovanadate (Na_3_VO_4_) and β-glycerol phosphate were prepared from Sigma Aldrich, USA. Tris–HCl, KCl, phosphoric acid, MDA, n-butanol, 2-thiobarbituric acid 5,5-dithio-bis-(2-nitrobenzoic acid (DTNB), ethylene diamine tetra acetic acid (EDTA), ethylene glycol tetra acetic acid (EGTA), urea, sodium dodecyl sulfate (SDS), glycerol, 2-mercaptoethanol (2-ME) and dry skim milk were purchased from Merck, Germany. Pierce™ ECL western blotting substrate were obtained from Thermo Fisher Scientific, USA. Protein Assay Kit II and polyvinylidene difluoride (PVDF) were bought from Bio Rad, USA. Stigmas of *C. sativus *was provided by Novin Saffron (collected from Ghaen, Khorasan Province, Northeast of Iran) and crocin was extracted from this herbal medicine based on the method which described by Hadizadeh *et al*, 2010 (48). The purity of the crocin was more than 97% as evaluated by HPLC and UV-visible spectrophotometry (48).

***Animals***

Adult male Wistar rats with 220±20 g were provided by the Animal Center, School of Pharmacy, and Mashhad University of Medical Sciences. Four rats were acclimatized per cage and maintained at 25±2 °C, with a 12-hr dark–light cycle with free access to food and water. Animals were allowed to habit to their surrounding environment for 1 week prior to the start of the experiments. The animal use and all procedures study were done according to Ethical Committee Acts of Mashhad University of Medical Sciences, Mashhad, Iran, under license number 941770. Animal studies are reported in compliance with the ARRIVE guidelines. 

***Experimental design and animal grouping***

In this study, in the first step, animals were randomly assigned to 5 treatment groups (with 6 rats/group) and were orally (gavage) administrated to different doses of BPA. These groups are including:

Group 1: Low dose of BPA (0.5 mg/ kg)

Group 2: Medium dose of BPA (5 mg/kg) (NOAEL, no-observed-adverse-effect level) (22)

Group 3: High dose of BPA (50 mg/kg) 

Group 4: High dose of BPA (100 mg/kg) 

Group 5: Sesame oil as control 

BPA was administered by oral route similar to human exposure through plastic and resin based food containers. BPA was dissolved in pure ethanol and diluted in sesame oil as the vehicle. The concentration of ethanol in final solution was 0.5% which has no adverse effects in animals (49). The exposure time was between 10:00 and 11:30 am per day and the duration of the exposure was 28 days. The animals’ body weight was checked weekly to adjust the dosage of BPA. After determination of the proper dose of BPA that induced memory impairment, in the next step, groups 6, 7, and 8 were designed as below (50):

Group 6: BPA (100 mg/ kg) + Crocin 10 mg/kg (IP) 

Group 7: BPA (100 mg/ kg) + Crocin 20 mg/kg (IP) 

Group 8: BPA (100 mg/ kg) + Crocin 40 mg/kg (IP) 

Group 9: Crocin 40 mg/kg (IP) 

Animals in all mentioned groups were treated daily for 28 days. During day 24-28 of the treatments, the acquisition test was done and on day 29, the probe test was performed. Details of the tests to evaluate memory function have been explained below. Twenty-four hours after finishing the treatment, the animals were sacrificed by decapitation in the accordance with the rules as considered by Ethical Committee Acts of Mashhad University of Medical Sciences. The hippocampi were dissected and aliquots the samples were stored at -80 ^°^C until use. 

***Morris water maze test***

The water maze apparatus comprised of a black circular pool with a diameter (136 cm in diameter×60 cm in height) divided into 4 equal quadrants defined by the cardinal directions (N, E, S, and W) was filled with water (22±1 °C) to a depth of 25 cm. A black plexiglass platform with a diameter of 13 cm was located 2 cm beneath the water surface in the center of one of the quadrants. Some constant visual cues visible to the rats were hung on the walls around the water maze apparatus. The testing room was dimly illuminated and temperature of the room was controlled and kept at (22±1 °C). A video camera which was connected to a computer was mounted above the apparatus to monitor the position of each rat and analyse the collected data (51, 52).

***Acquisition test***

During the training trials (day 24-28 of the treatments), in the acquisition test protocol, each rat done 4 trials per block daily for 5 days. Rats were allowed to swim for 60 sec to find the hidden platform and then were stayed on the platform for 15 sec. If a rat could not find the platform within the maximum time of 60 sec, it was manually placed on the platform for 15 sec. The escape latency (sec) to find the hidden platform, the escape pathlength (cm, distance travelled to the hidden platform), and the swim speed (cm/sec) were automatically recorded using the video camera (51, 52). 

***Probe test***

On the sixth day of the test (24 hr after the last training trial) the probe test consisted of 4 trials per block was performed. A 60 sec trial was done for animals when the platform was removed to test the spatial memory. The starting point for each animal was randomly selected. The total time spent in the goal quadrant and travelled distance during the probe trial was recorded with a computer-based video tracking system (51). 

***Sample collection***

At the end of the Morris water maze test, rats were decapitated and the hippocampus was separated. Then

the samples were snap-frozen in liquid nitrogen and

stored at -80 °C. For each test brain homogenate was

prepared using the protocol described below. 

***Measurement of lipid peroxidation***

***Lipid peroxidation was determined in hippocampus by measurement of the malondialdehyde (MDA) content based on the the protocol described by Mihara*** *et al*. with changes (53). Tissues were weighed and homogenized in ice-cold 0.15 M KCl (potassium chloride) solution to prepare 10% (w/v) homogenates. After that, 0.5 ml of tissue homogenate was mixed with 3 ml of 1% (w/v) phosphoric acid and 1 ml of 0.6% (w/v) 2-thiobarbituric acid (TBA) and the mixture was heated in boiling water bath for 45 min. In this step, reaction of MDA with TBA produces thiobarbituric acid reactive substance (TBARS) which has pink colour with maximum absorbance at 532 nm. After cooling to room temperature, n-butanol was added to the mixture and vortexed well for 1 min. Centrifuge was done at 4000 g for 10 min. The organic layer (n-butanol) was removed and transferred to a fresh tube and the absorbance of pink colour was recorded at 532 nm using a spectrophotometer (Jenway 6105 UV/Vis, UK). The level of MDA in each sample was reported as nmol/g tissue.

***Determination of reduced glutathione (GSH) content***

The procedure described by Moron *et al.* was applied to measure the GSH content as a non-enzymatic anti-oxidant agent in hippocampus (54). Tissues were washed with ice cold saline and homogenized in ice-cold 0.1 M phosphate buffer (pH=7.4). The equal volume of brain homogenate was mixed with 0.5 ml of the trichloroacetic acid and centrifuge was done at 10,000 *g* for 10 min to remove protein precipitate. Then 2.5 ml of 0.3 M phosphate buffer (pH=8.4) and 0.5 ml of 0.04% (w/v) 5,5’-dithio-bis(2-nitrobenzoic acid (DTNB) solution were added to 0.5 ml of the supernatant fraction containing GSH to produce the yellow coloured 5′-thio-2-nitrobenzoic acid. The absorbance of the solution was determined at 412 nm within 5 min using the spectrophotometer (Jenway 6105 UV/Vis, UK) and the GSH content was expressed as nmol/g tissue. 

***Western blotting***

This test was done according to the methods, which completely were described before (21, 22). Briefly, total protein was extracted from hippocampi tissues of each group in lysis buffer containing 50 mM Tris–HCl (pH=7.4), 2 mM EDTA, 2 mM EGTA, 10 mM NaF, 1 mM Na_3_VO_4_, 10 mM β-glycerol phosphate, 0.2% w/v sodium deoxycholate, 1 mM PMSF, 10% v/v 2-ME, and 2 μl protease inhibitor cocktail and protein concentrations of the supernatant were measured using Bio-Rad Protein Assay Kit based on the manufacturer’s instructions. Then the equal volumes of the supernatants were diluted in SDS sample buffer and heated in boiling water for 5 min. Protein samples were loaded on 12% SDS-PAGE gels, run for 90 min at 120 V and transferred to polyvinylidene difluoride (PVDF) membranes. Then, the membranes were blocked in Tris buffered saline–Tween 20 (TBST) containing 5% dry skim milk for 2 hr at room temperature. Primary antibodies including: NMDA rabbit monoclonal antibody (Cell Signaling, #4205), AMPA rabbit monoclonal antibody (Cell Signaling, #2460) and β-Actin mouse monoclonal antibody (Cell Signaling, #3700) were used according to manufacturer’s protocols. Then, blots were washed with TBST and incubated with a horseradish peroxidase-conjugated secondary antibody (Cell Signaling, #7076, #7074) at 1:3000 dilutions for 1 hr. Membranes were then developed using Pierce™ ECL western Blotting Substrate and Alliance 4.7 gel doc (UK). All intensities of the chemiluminescence bands were normalized against corresponding beta actin intensities and quantified using UVI-BandMap software (UVtec, UK).

***Statistical analysis***

The data were expressed as the mean±standard deviation (SD). In all the tests, technical replicates were used to ensure the reliability of single values. Analysis of data was performed by One-way or two- way Analysis of Variance (ANOVA) followed by Tukey *post hoc* test. All data were processed by GraphPad Prism version 6.00 for Windows, GraphPad Software, La Jolla California USA. *P* values less than 0.05 (*P*<0.05) were considered as statistically significant. 

## Results

***Effect of crocin on memory dysfunction induced by BPA: Morris water maze test***

**Figure 1:**
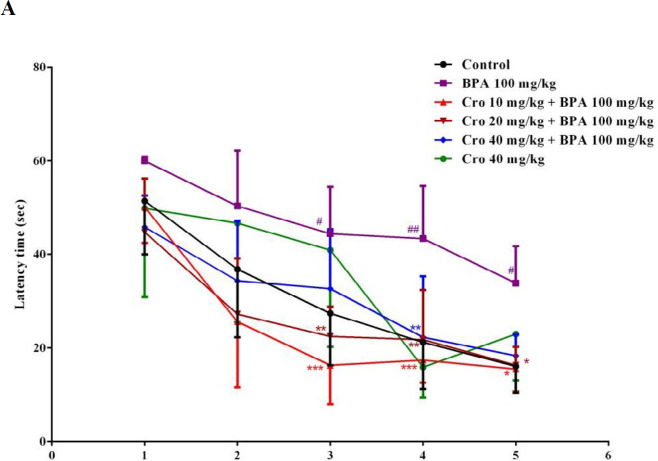
The effects of crocin (Cro) on the escape latency (sec) from day 1 to day 5 of training trial (A) and the time (sec) spent in the target quadrant (B) to find the hidden platform in the bisphenol A (BPA) exposed rats in Morris water maze test. Data are expressed as the mean±SD (n=6 animals per group). #*P*<0.05, ##*P*<0.01 between the control group and the BPA group, *P<0.05, **P<0.01 and ***P<0.001 between the Cro+BPA group and the BPA group. Data were analyzed through one-way or two–way ANOVA coupled with Tukey-Kramer multiple comparisons test

**Figure 2:**
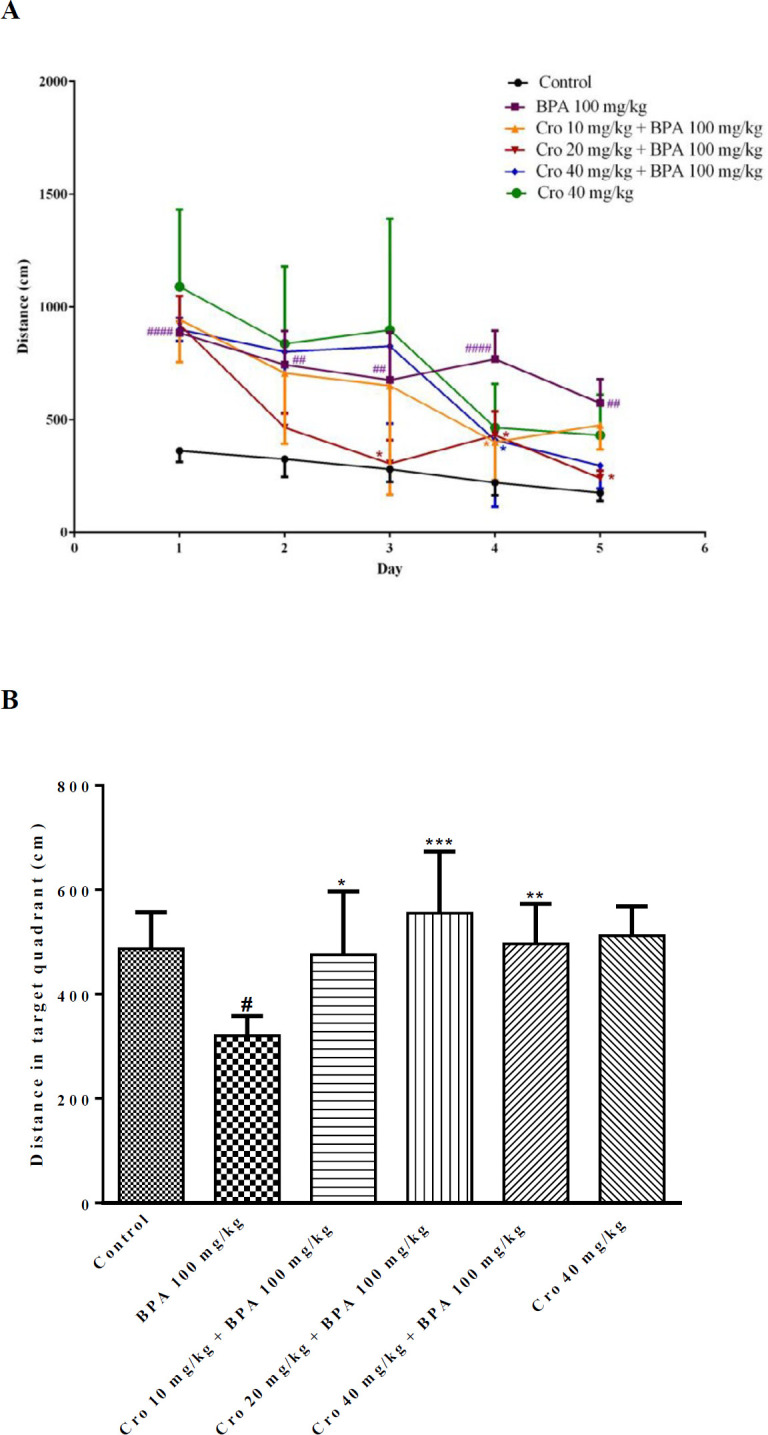
The effects of crocin (Cro) on the traveled distance (cm) from day 1 to day 5 of training trial (A) in the target quadrant (B) to find the hidden platform in the bisphenol A (BPA) exposed rats in Morris water maze test. Data are expressed as the mean±SD (n=6 animals per group). #*P*<0.05, ##*P*<0.01, ###*P*<0.001 between the control group and the BPA group, *P<0.05, **P<0.01 and ***P<0.001 between the Cro+BPA group and the BPA group. Data were analyzed through one-way or two- way ANOVA coupled with Tukey-Kramer multiple comparisons test

**Figure 3:**
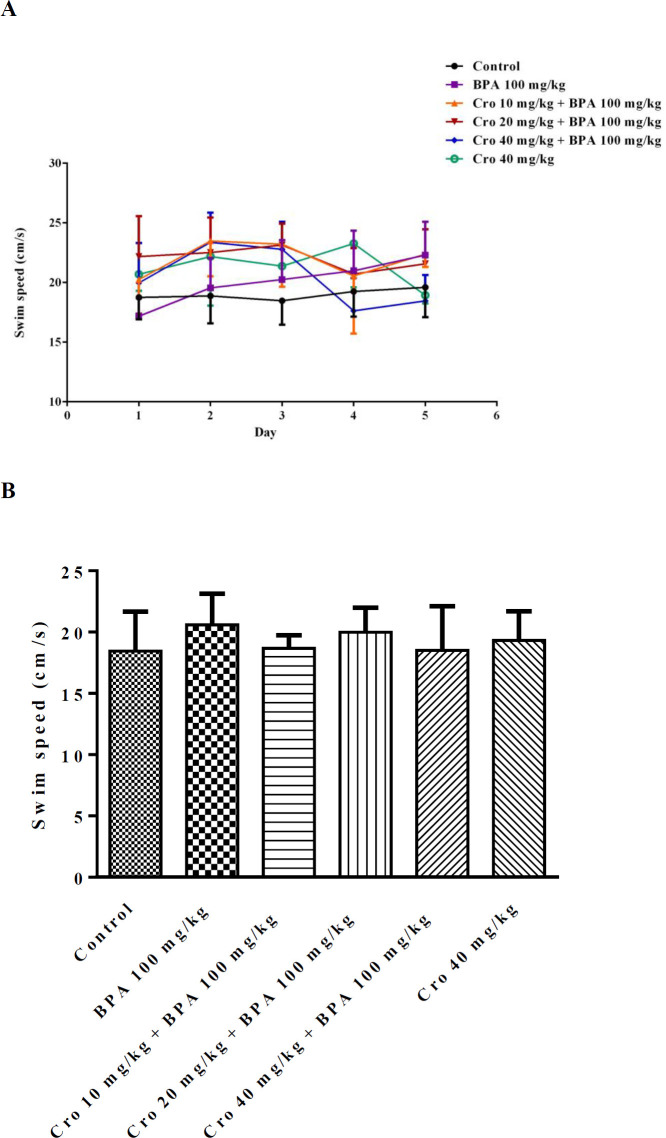
The effects of crocin (Cro) on the swimming speed (cm/s) (A) from day 1 to day 5 of training trial and on the day of probe test (B) after removing the platform in the bisphenol A (BPA) exposed rats in Morris water maze test. Data are expressed as the mean±SD (n=6 animals per group)

In the MWM test, according to results which included in supplementary data, BPA (100 mg/kg) markedly elevated the time for finding the platform in training trial. Also, administration of BPA (100 mg/kg) significantly decreased the time spent in the target quadrant in probe trial, so, this dose was selected for the continue of our study. Two-way ANOVAs exhibited that the average escape latency (sec) meaningfully changed following BPA exposure × training days (*P*<0.05) compared to that of control group (on day 3, BPA 44.4±10.0 sec, control, 27.4±11.1 sec; on day 4, BPA 43.4±11.2 sec, control, 21.2±10.0 sec; on day 5, BPA 33.9± 7.8 sec, control, 16.1±5.6 sec, respectively). Travelled distance (cm) was significantly longer than that of other groups following interactions of BPA exposure × training days compared to control group (*P*<0.05) on the all days’ acquisition trial (on day 1, BPA, 885±6.7 cm, control, 362.1±5.0 cm; on day 5, BPA 574±10.5 cm, control, 176±3.6 cm). Exposure to BPA 100 mg/kg/day, markedly attenuated the time spent in the target quadrant (sec) (BPA, 16.7±2.6 sec, control, 26.2±5.4 s) and the travelled distance (cm) (BPA, 320±3.8 cm, control, 487.2±7.0 cm) on day 6 when compared to that of the controls (*P*<0.05) (Figure 1B and Figure 2B). The escape latency (sec) and the travelled distance (cm) in BPA group during final 3 days of acquisition trial were abolished by crocin exposure × training days (*P*<0.05) at all doses compared to that of BPA only exposed group ( Figure 1A, Figure 2A). Administration of crocin (10, 20 and 40 mg/kg) markedly elevated the travelled distance (cm) and the time (sec) spent in the target quadrant during the probe trial on day 6 of test when compared to BPA exposed group (Figure 1B, Figure 2B). Also, there was no significant difference of swimming speed among groups in the MWM test during 5 days of training and probe trial (Figure 3A, B).

***Effect of crocin and BPA on GHS content and MDA level in rat hippocampus***

**Figure 4:**
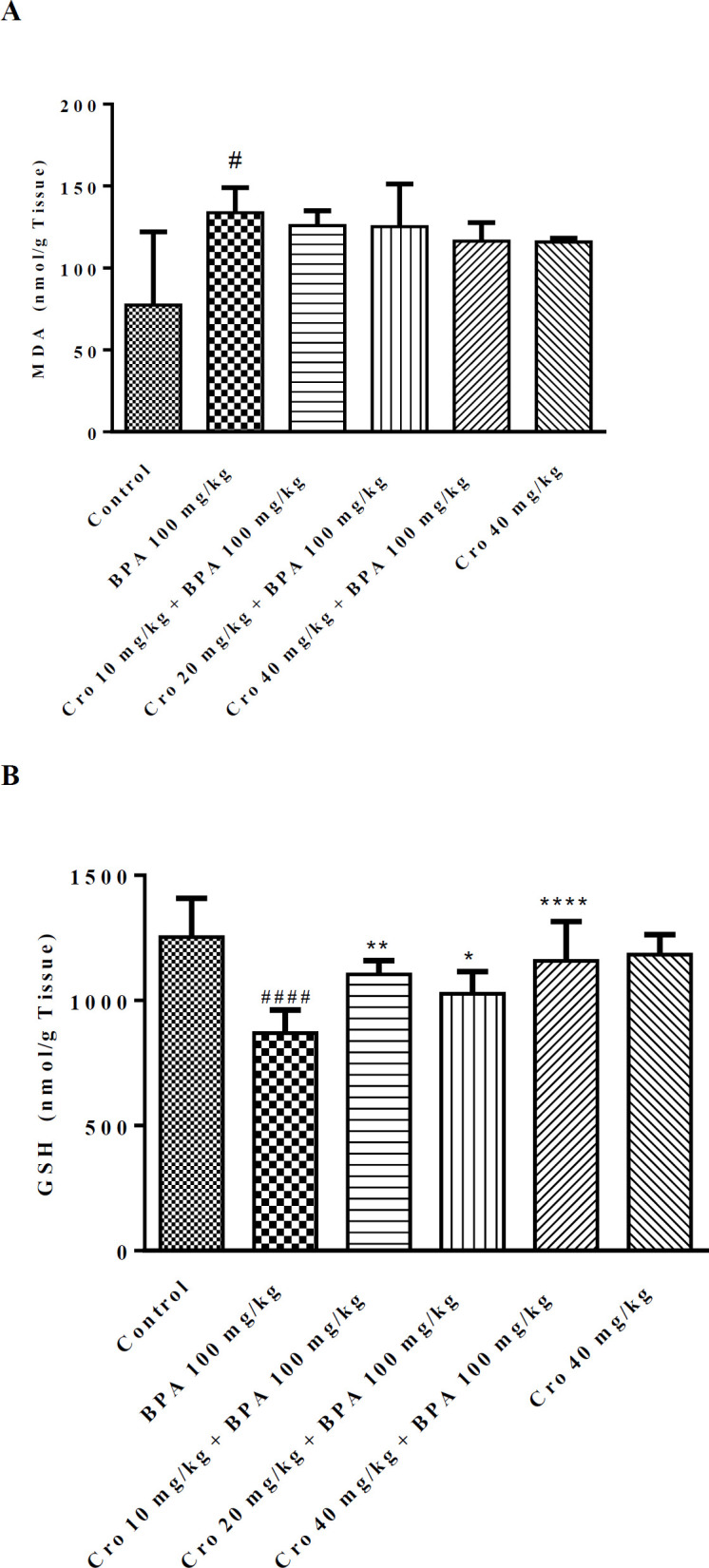
The effects of crocin (Cro) on malondialdehyde (MDA) (A) and glutathione (GSH) (B) levels in the hippocampus of bisphenol A (BPA) exposed rats. Data are expressed as the mean±SD (n=6 animals per group). #*P*<0.05 and #### *P*<0.0001 compared to control group, *P<0.05, **P<0.01 and ***P<0.0001 compared to BPA group. Data were analyzed through one-way ANOVA coupled with Tukey-Kramer multiple comparisons test

In order to examine the influence of crocin on BPA-induced oxidative stress, the levels of GSH and MDA were measured in the rat hippocampus. As showed in Figure 4A, BPA exposure increased MDA generation in the hippocampus as compared to the control group (*P*<0.05, BPA, 133.7±15.4 nmol/g tissue; control, 77.4±44.6 nmol/g tissue). Compared with the control, the GSH content in the hippocampus was meaningfully decreased by BPA 100 mg/kg/day (Figure 4B, *P*<0.0001, BPA, 868.8±92.7 nmol/g tissue; control, 1254.0±154.4 nmol/g tissue). Crocin at 10, 20 and 40 mg/kg /day markedly increased the level of GSH content whereas did not change the levels of MDA when compared to BPA group. 

***Effect of crocin and BPA on the level of NMDA and AMPA receptors proteins in rat hippocampus***

**Figure 5:**
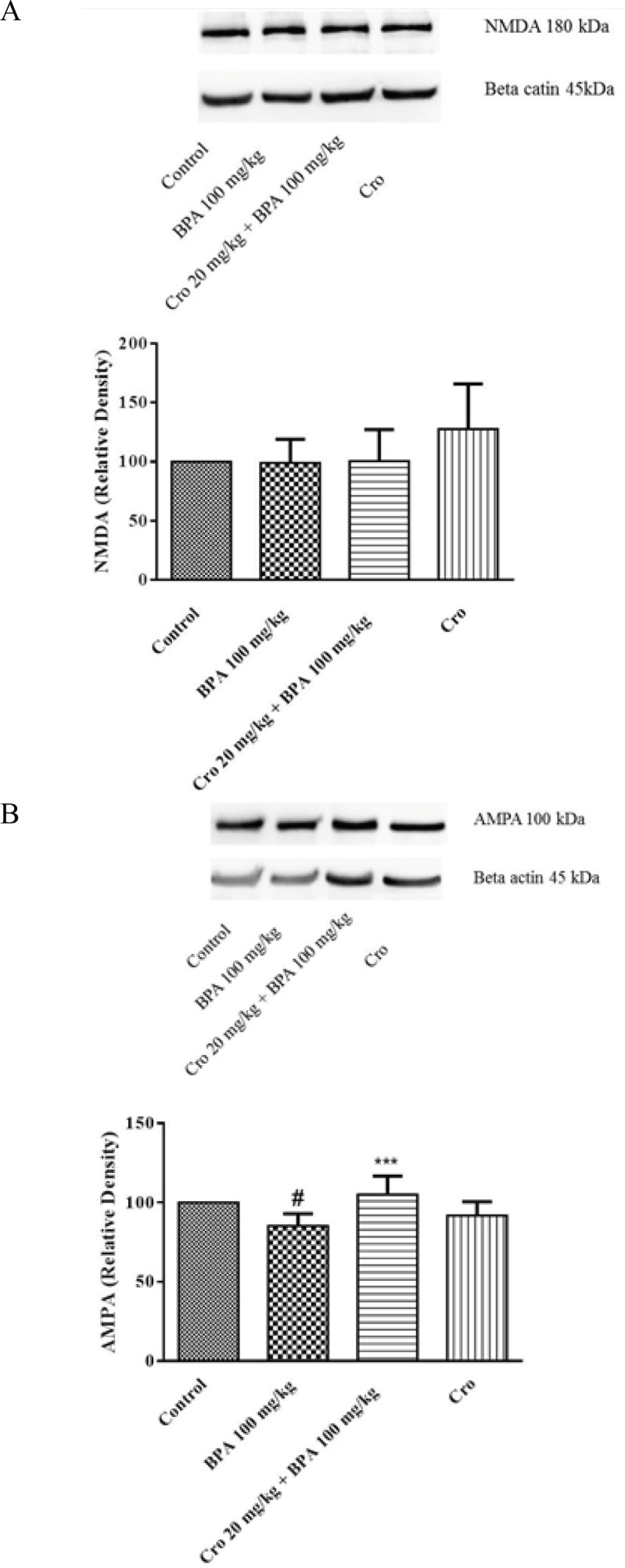
The effects of crocin (Cro) on the protein expressions of NMDA (A) and AMPA (B) receptors in the hippocampus of the bisphenol A (BPA) exposed rats. Data are expressed as the mean±SD (n=4 animals per group). β-Actin was used as the loading control. #*P*<0.05 compared to control group, ***P<0.001 compared to BPA group. Data were analyzed through one-way ANOVA coupled with Tukey-Kramer multiple comparisons test

***In order to clarify the mechanisms underlying the effect of crocin on changes of behaviors and synapse morphology induced by BPA, ***synaptic NMDA (NMDA 2A) and AMPA receptors (GluA 2/3/4) were analyzed by Western blot. The results exhibited that the expression of the synaptic protein AMPA was significantly down-regulated following exposure to BPA 100 mg/kg/day (*P*<0.05), exposure as compared to the control group; whereas no significant difference in the levels of NMDA protein were determined in the hippocampus. The results demonstrate that glutamate receptor in the adult male hippocampus is affected by BPA and consequently induces memory impairment (Figure 5). As observed in Figure 5, administration of crocin 20 mg/kg/day markedly elevated the hippocampal expression of AMPA when compared to BPA exposed rats (*P*<0.001). 

## Discussion

This study is the first to investigate the effects of crocin on the sub-acute exposure to BPA in adult rats, which impairs spatial learning and memory via oxidative stress and changing the protein levels of receptors involved in the hippocampus synaptic plasticity. Many chemical agents which enhance cognitive functions cause health side-effects and replacing them with herbal extracts or related active compounds may be an ideal choice. Our results exhibited the protective effects of crocin on BPA induced learning and memory impairments in MWM tasks. Also, crocin elevated the GSH content and the level of AMPA receptor protein in hippocampus. 

It is well known that the hippocampus is the most important region in synaptic plasticity and memory formation which its lesions produce severe amnesia for certain memories in both humans and other mammals (9). Brain has a low storage capacity for ROS accumulation and a high capacity for lipid peroxidation, so the brain cells are susceptible for oxidative stress mediated damage (55). Persistent increase in oxidative stress can negatively affect learning and memory through alteration in neurogenesis and dendritic structures in the hippocampal formation (56) and promotes cell injury and death in many neurodegenerative disorders. Toxicological studies have reported that oxidative stress is one of the important factors of BPA toxicity in the different parts of the brain and other organs like liver and epididymal sperm (20, 22, 57). Anti-oxidant agents have been shown to improve cognitive deficits (58-60). BPA penetrates the brain and induces oxidative stress in different organs (61), so the application of anti-oxidants, which can transfer, to the brain would be a possible therapeutic approach against BPA induced oxidative damage in sensitive sites. In support of this view, N-acetylcysteine (62), vitamin C (63), and melatonin (55) reversed cognitive dysfunction and oxidative stress induced by BPA exposure in the brain of rats. In the present study, crocin significantly modulated the redox state in the hippocampus of BPA-treated rats verified by an increase in the levels of GSH. Elevation of GSH level, increase the activity of anti-oxidant enzymes and reduction of lipid peroxidation have been considered as different functions of crocin to reduce oxidative stress (64). Spatial cognitive abilities improvement by crocin was demonstrated in chronic cerebral hypoperfusion (59) or chronic stress (65) of rats. The possible crocin mechanisms are largely related to its anti-oxidant effects. Crocin could attenuate cognitive deficits in D-galactose aging model, and the neuroprotective effect was mediated by alleviation of oxidative stress and inflammation (51). Modulation of oxidative stress, autophagy and apoptosis markers are involved in the reversal effect of crocin on memory deficits (66, 67). Crocin in our investigation was capable of ameliorating BPA-induced oxidative stress by enhancing the levels of reduced GSH in the hippocampus; however, its effects on lipid peroxidation were incomplete suggesting the higher doses of crocin might be applied in order to show full protection.

In the MWM, co- administration of crocin with BPA reduced the latency time to find hidden platform during training days (day 24-28 of the treatments) when compared to BPA received group. Additionally, in probe trials, the spent time in target quadrant markedly elevated in crocin + BPA groups in comparison to BPA only received animals. Most of the studies have found effects of perinatal BPA exposure on brain and behaviors later in life (68, 69) and the effects of BPA exposure on memory in adults have not completely determined. 

Xu *et al*. showed that exposure to BPA (0.4 and 40 mg/kg/day) during adult hood for 12 weeks markedly elevated latency to find the platform in the MWM test consequently altered characteristic differences between male and female mice including spatial learning and memory and passive avoidance memory (28). In another study, exposure to BPA 40 µg/kg/day for 8 weeks significantly impaired the spatial learning and memory of the male mice but not the female mice (27). Eilam-Stock *et al*. showed formation of new memories markedly was impaired following exposure to a single dose of BPA 40 µg/kg through alteration of neural plasticity processes in the adult male rat brain (26). Kim *et al*. found that high-dose (20 mg/kg/day) but not low-level (1 mg/kg/day) BPA exposure for 2 weeks in young adult mice induced higher latencies in the MWM (70). In our study, administration of BPA (100 mg/kg) to adult rats markedly elevated the time for finding the platform in training trial and induced memory impairment. Additionally, administration of BPA significantly attenuated the time spent in the target quadrant in probe trial which confirmed memory dysfunction in accordance with previous reports which mentioned above.

 Neuroprotective effects of *C. sativus* extracts preparation and its pharmacologically active ingredient crocin were demonstrated in several neurological diseases including convulsion (71), anxiety (72), and depression (41, 42). In the MWM test, crocin markedly reduced the escape latencies prolonged by BPA and increased the spent time in the target quadrant. These data are consistent with previous reports which crocin exhibited protective effects against memory impairment-induced by chronic cerebral hypoperfusion (45), D-galactose (51) and scopolamine (46) in animal models.

Concomitant with the altered oxidative stress in the hippocampus, BPA administration significantly attenuated the level of AMPA receptor (GluA 2/3/4) but did not change the level of NMDA receptor (NMDA 2A). NMDA and AMPA receptors are involved in the mediation of the fast synaptic transmission between neurons controlling synaptic plasticity and formation of memory (73, 74). The present results are in part in agreement with the obtained data that BPA treatment (40 mg/kg) for 12 weeks impaired spatial memory and down-regulated expressions of synaptic NMDA receptor subunit NR1 and AMPA receptor subunit GluR1 in the hippocampus of the adult mice brain (28). It was recently reported that postnatal treatment of male Sprague–Dawley rats with BPA (1 mg/kg/day, 21–24 to 49–52 day) resulted in significant neurocognitive disorder in synaptic plasticity and spatial memory and reduced NMDA receptor 2A (NR2A) and AMPA receptor subunit GluR1 expression contributing to memory deficits (75). In this regard, crocin in the present study protected against learning and memory impairments–induced by BPA and increased expression of AMPA in the hippocampus of the BPA-exposed rats, demonstrating that the memory improvement effect of crocin could involve the control of AMPA expression. Ethanol inhibits NMDA receptor-mediated responses and consequently blocks induction of LTP in hippocampal neurons. These effects of ethanol could be antagonized following treatment by crocin (76). Recently we reported a positive effect of crocin on the memory impairment of the adult male rats induced by hyoscine following 4 weeks exposure. No significant changes were observed in the levels of NMDA and AMPA receptors in rat hippocampus. It can be concluded that memory improvement effect of crocin is mediated through other important pathways involved in memory formation and neuronal plasticity (77). 

## Conclusion

Our study provided valuable information about the effects of exposure to BPA on spatial learning and memory function in adult male rats and protective effect of crocin. We suggested that the impaired memory in the adult males may be related to induction of oxidative stress and down-regulation of the synaptic receptor AMPA in hippocampus following BPA exposure. Crocin as an important constituent of *C. sativus* improved learning and memory and up-regulated hippocampal AMPA receptors. Our findings exhibited the underlying mechanism of crocin and confirmed this compound as a new candidate treatment of impairing cognitive disorders including Alzheimer’s disease. 
